# Effect of high amylose maize starches on colonic fermentation and apoptotic response to DNA-damage in the colon of rats

**DOI:** 10.1186/1743-7075-6-11

**Published:** 2009-03-07

**Authors:** Richard K Le Leu, Ying Hu, Ian L Brown, Graeme P Young

**Affiliations:** 1 Flinders Centre for Cancer Prevention and Control, Flinders University of South Australia, Bedford Park, South Australia 5042, Australia

## Abstract

**Background:**

We investigated in rats the effects of feeding different forms of high amylose maize starches (HAMS) rich in resistant starch (RS) to understand what the implications of RS heterogeneity might be for colonic biology, including innate cellular responses to DNA-damage.

**Methods:**

A range of maize starches were compared: digestible cornstarch (Control), HYLON^® ^VII, Hi-maize^® ^1043, Hi-maize^® ^240, Hi-maize^® ^260 and NOVELOSE^® ^330. Included in the comparison was Cellulose. End-points after 4 weeks included: pH, short chain fatty acids (SCFA) levels, colonic epithelial cell kinetics and apoptotic response to carcinogen 'azoxymethane' in the colonic epithelium.

**Results:**

The RS diets significantly increased SCFA and reduced pH in caecal content and faeces. Hi-maize 260 resulted in the highest butyrate concentrations. All RS diets prevented the mucosal atrophy as seen in the rats fed the Control diet. Epithelial cell turnover was increased in the Control and Cellulose groups compared to the Hi-maize 260, HYLON VII and NOVELOSE 330 groups (P < 0.01). The apoptotic response to azoxymethane was higher only in the Hi-maize 260 group compared to the Control group (P < 0.01). Butyrate correlated positively with the apoptotic response (P < 0.01).

**Conclusion:**

The consumption of RS elicits a range of beneficial physiological and protective effects associated with the fermentation of RS. Increased production of butyrate seems a likely explanation by which RS enhances the apoptotic response to carcinogen-induced DNA damage which is consistent with the proposed role of this SCFA in promoting a normal cell phenotype and preventing the development of abnormal cell populations.

## Background

Resistant starch (RS) is defined as a component of dietary starch that is not absorbed in the small intestine of healthy individuals and thus reaches the colon undigested, similar to dietary fibre [1]. Evidence is mounting to suggest that RS is a protective agent against several serious gastrointestinal disorders [2] including that of colorectal cancer [3,4]. The mechanism for protection may be associated with metabolic products of anaerobic bacterial fermentation of RS. Fermentation of RS produces short-chain fatty acids (SCFA) which lower luminal pH; increase bacterial biomass and faecal bulk; and modify the composition of the microbiota, especially by stimulating the growth of beneficial bacteria including bifidobacteria and lactobacilli [5]. Butyrate, one of the principal colonic SCFA has generated the most interest and may be protective against colorectal cancer [6,7]. Butyrate has been shown to be the primary energy source for the colonic epithelium [8]; it inhibits the growth of cancer cells *in vitro *and forces a more normal differentiated phenotype [6]. In addition, it is a potent pro-apoptotic agent [9] as it removes genetically disordered cells which help to counteract the biological consequences of genomic instability. Colonic production of butyrate by fermentation is associated with reduced tumour mass in an animal model, provided that fermentation is active in distal colon [10]. More recently in an experimental animal studies, RS feeding was associated with the generation of increased butyrate levels in the colon as well as protecting against carcinogen-induced colon tumourigenesis [11,12].

Further clarification of the health benefits of RS is, however, made difficult by the fact that it is heterogeneous in nature with the many different forms varying in their physicochemistry, digestibility, fermentability and fibre content. Types of RS have been traditionally classified into four main types based upon structural considerations and bacterial fermentability [1]. RS1 includes physically entrapped starch within whole plant cells and food matrices (e.g. coarsely milled grain). RS2 consists of native starch granules that are highly resistant to digestion by α-amylases (e.g. green banana, HAMS). RS3 comprises retrograded starches, formed when starchy foods are cooked and cooled. RS4 comprises chemically modified starches (e.g. esterified starches) where the modification interferes with the amylolytic activity of digestive enzymes. Apart from these structural differences, different sources of RS may vary according to proportion of nondigestible starch, dietary fibre content, starch granule size and other physico-chemical differences that might result in different levels of digestibility and fermentation patterns [13]. This might lead to different luminal conditions and hence variations in epithelial biological responsiveness. It has also been recently shown that there can be an important synbiotic interaction with colonic microbiota resulting in altered epithelial response to carcinogen-induced genomic lesions in the colon [5]. Because of the diversity and forms of RS they cannot be expected to all perform in the same manner physiologically [14]. As a consequence, different forms of RS might vary considerably in their effects on colonic biology and the consequences of that for colonic disorders such as cancer.

The aim of the current study was to investigate the effects of feeding high amylose maize starches (HAMS) that differed in their RS content, dietary fibre content and degree of processing on colonic fermentation and implications for colonic biology. We have explored their effects on luminal conditions, namely SCFA concentrations and pH, and the relationships to certain epithelial events potentially regulated by luminal conditions: specifically colonic epithelial proliferation and apoptotic response to a DNA-damaging agent. The study focused on RS2 and RS3 derived from HAMS and included native and hydrothermally (or heat-moisture) treated forms. Hydrothermal processing is a means of significantly increasing the dietary fibre content of HAMS. The starches examined were as follows: Hylon VII which is a RS2 type starch and is the base starch for the preparation of Hi-maize 240 and Hi-maize 260. Hi-maize 1043 is a RS2 type starch that has been hydrothermally prepared from HAMS, as reported by Bird et al. [15]; although made from a different base HAMS, the Hi-maize 1043 and Hi-maize 260 have been made using similar hydrothermal methodology. Novelose 330 which is a retrograded RS3 generated from the hydrolysed products of corn starch, [16] and has not been previously reported in terms of apoptotic response to a DNA-damaging agent.

## Methods

### Animals and diets

A total of 84 male Sprague-Dawley rats, 5 weeks of age, were obtained from the Animal Resource Centre, Perth, Western Australia. Animals were divided randomly into seven experimental groups of equal bodyweight and housed three per plastic cage in an animal holding room under controlled conditions of 22 ± 1°C (SE), 80 ± 5% humidity, and 12 h light/dark cycle. Animals were given free access to water and weighed weekly throughout the study. Rats were fed experimental diets for four weeks.

Five different RS forms were used in the study. All of the forms of RS are derived from HAMS and the amounts of RS and total dietary fibre (TDF) and information on what treatment the starch has undergone are shown in Table 1. The hydrothermal process was conducted using HAMS under conditions where the starch had a moisture content of 25% and it was heated to 125°C for 120 min. The experimental diets were modified forms of the AIN-76a standard for purified diets for rats and mice [17]. Each group of animals was fed an experimental diet based on the control diet (Table 2). Choline, methionine, minerals, and vitamins were added as previously. The first group "Control" consumed a diet containing no added fibre or RS. The second group "Cellulose" was fed α-cellulose (Sigma Chemical Company, St Louis, MO) at a level of 50 g/kg diet (equivalent to 5% total dietary fibre). The remaining groups were fed various forms of HAMS at a level of 200 g/kg diet. These HAMS are all available commercially and are excellent sources of RS but differ widely in their physicochemical characteristics. The third group "HYLON VII" was fed HYLON VII. The fourth group "Hi-maize 1043" was fed Hi-maize 1043. The fifth group "Hi-maize 240" was fed Hi-maize 240. The six group "Hi-maize 260" was fed Hi-maize 260. The seventh group "NOVELOSE 330" was fed NOVELOSE 330. The different forms of HAMS were supplied by National Starch and Food Innovation (Bridgewater, New Jersey, USA) and were added to the diets at the expense of an equal amount of digestible cornstarch.

**Table 1:** Treatment procedure, dietary fibre and resistant starch levels of the different RS forms^1,2^

	**HYLON VII**	**Hi-maize 1043**	**Hi-maize 240**	**Hi-maize 260**	**NOVELOSE 330**
Treatment	none	hydrothermal	hydrothermal	hydrothermal	retrogradation
RS type	RS2	RS2	RS2	RS2	RS3
RS amount (%)^1^	48	50	53	46	47
Total Dietary Fibre (%)^2^	18	62	40	60	36

^1 ^The RS values were determined on a dry weight basis for the different HAMS by AOAC 2002.02 method.

^2 ^The total dietary fibre (TDF) were determined on a dry weight basis for the different amylose maize starches by AOAC Method 991.43.

**Table 2:** Composition of experimental diets (g/100 g diet)^1^

**Ingredient**	**Control**	**Cellulose**	**HYLON VII**	**Hi-maize 1043**	**Hi-maize 240**	**Hi-maize 260**	**NOVELOSE 330**
Casein	20.00	20.00	20.00	20.00	20.00	20.00	20.00
Corn starch	46.15	41.15	26.15	26.15	26.15	26.15	26.15
α-cellulose	-	5.00	-	-	-	-	-
HYLON VII	-	-	20.00	-	-	-	-
Hi-maize 1043	-	-	-	20.00	-	-	-
Hi-maize 240	-	-	-	-	20.00	-	-
Hi-maize 260	-	-	-	-	-	20.00	-
NOVELOSE 330	-	-	-	-	-	-	20.00
Corn oil	18.00	18.00	18.00	18.00	18.00	18.00	18.00
Sucrose	10.95	10.95	10.95	10.95	10.95	10.95	10.95
dl-Methionine	0.3	0.3	0.3	0.3	0.3	0.3	0.3
Choline	0.1	0.1	0.1	0.1	0.1	0.1	0.1
Mineral mix^1^	3.5	3.5	3.5	3.5	3.5	3.5	3.5
Vitamin mix^1^	1.0	1.0	1.0	1.0	1.0	1.0	1.0

^1 ^AIN-76 vitamin and mineral mixtures [17]

### Experimental procedure

After three weeks on experimental diets rats were housed temporarily in metabolic cages and faecal output was measured for 24 hours. Fresh faecal samples were collected from each rat during the last 3 d of the experimental period by gently handling the rats until they produced a faecal sample. For faecal pH, fresh faeces were homogenized in 3 volumes of saline and the pH recorded (TPB, digital pH meter, model 1852 mV). Fresh faeces were diluted in 3 volumes of internal standard solution (heptanoic acid, 1.68 mmol/L) and stored at -20°C for later analysis of SCFA concentrations.

On the final day of the experimental period, (after 4 weeks on the diet), each rat was administered a single i.p. injection of azoxymethane (AOM), 10 mg/kg body weight, (Sigma Chemical) to induce genomic damage and initiate damage-response events including apoptosis [18]; rats were killed by CO2-induced narcosis 6 h later, the time of maximal apoptotic response [19]. The entire colon was rapidly removed and divided into proximal and distal portions; the limit of the proximal portion was defined by the "herring bone" pattern. These were flushed clean with ice-cold saline, and a segment (2 cm) was taken from the rectal end of the distal portion. This segment was placed in 10% buffered formalin for 24 h, then washed and stored in 70% ethanol. The caecum was excised, weighed, and a known weight of digesta placed in 3 volumes of saline for pH measurement; a known weight of digesta was also diluted in 3 volumes of internal standard solution (heptanoic acid, 1.68 mmol/L) and stored at -20°C for analysis of SCFA.

The Flinders University of South Australia Animal Welfare Committee approved all experimental procedures.

### Apoptosis in colonic epithelium

Colon sections (0.5 cm × 0.5 cm) in 70% ethanol were cut from distal segments of the colon embedded in paraffin. Paraffin-embedded sections (5 μm) were stained with hematoxylin and evaluated under a light microscope for apoptotic cells. Apoptotic cells were identified in 20 randomly chosen intact crypts by cell shrinkage, presence of condensed chromatin and sharply delineated cell borders surrounded with a clear halo as reported previously [20]. The percentage of apoptotic nuclei (apoptotic index) was calculated as the mean number of apoptotic cells/crypt column multiplied by 100. The length of each crypt was determined along with the position of apoptotic cells.

### Cell proliferation in colonic epithelium

Proliferative activity of epithelial cells was measured using immunohistochemical staining with Ki-67 monoclonal antibody (PC-10 clone, Santa Cruz, USA). In brief, paraffin embedded sections were deparaffinized in xylene and rehydrated through graded ethanol solutions to distilled water. Antigen retrieval was carried out by heating sections in 0.1 M citrate buffer pH 6.5 for 1 hour in a pressure cooker. Endogenous peroxidase activity was quenched by incubation in 3% H2O2 in methanol for 5 minutes. Sections were than incubated overnight at room temperature with Ki-67 antibody diluted in 1:1000. Detection was by biotinylated secondary rabbit-anti-mouse polyclonal antibody in 1/200 (Dako) for 30 mins and avidin/biotinylated peroxidase complex (Signet Laboratories) incubating for 20 mins. Slides were visualized by incubating with 3'-diaminobenzamine substrate. Positive staining was noted by brown precipitate in the cell cytoplasm. In all cases, an independent observer unaware of the dietary treatments measured Ki-67 positive cells. Epithelial turnover was assessed as the Ki-67 positive cells per crypt column length.

### SCFA analysis

SCFA including acetate, propionate and butyrate were determined in the caecal content and faeces of rats as described previously. [5]

### Statistical analysis

Data are expressed as mean ± standard errors, and differences between means were analysed by one-way ANOVA. Differences were considered significant at P < 0.05. Statistical differences were then separated by Tukey multiple comparison test. The correlations among variables were analysed by Spearman's correlation test, a value of P < 0.05 was used as the criterion of significance. The statistical package SPSS version 14 software was used for all analyses.

## Results

### Bodyweights and food intake

No significant difference was observed in the final bodyweights between the rats fed the different experimental diets (Table 3). Rats fed NOVELOSE 330 had a higher food intake compared to the rats consuming the other RS containing diets.

**Table 3:** Effect of experimental diets on final bodyweight, food intake, faecal output, pH, caecal weight and caecal content weight^1^.

	**Control**	**Cellulose**	**HYLON VII**	**Hi-maize 1043**	**Hi-maize 240**	**Hi-maize 260**	**NOVELOSE 330**
Final bodyweight	369 ± 4	353 ± 8	345 ± 10	345 ± 11	341 ± 9	366 ± 8	359 ± 4
Food intake (g/d)	17.9 ± 0.8^bc^	18.0 ± 0.5^bc^	15.5 ± 0.7^a^	15.8 ± 0.7^ab^	15.4 ± 0.7^a^	15.6 ± 0.5^ab^	19.0 ± 0.4^c^
Faecal Output (g/d)	0.5 ± 0.1^a^	1.7 ± 0.1^b^	1.7 ± 0.2^b^	1.9 ± 0.2^b^	1.7 ± 0.2^b^	2.2 ± 0.2^b^	3.2 ± 0.3^c^
Faecal pH	7.4 ± 0.05^d^	7.1 ± 0.03^c^	5.7 ± 0.03^ab^	5.8 ± 0.05^b^	5.7 ± 0.04^ab^	5.8 ± 0.07^b^	5.5 ± 0.03^a^
Caecal pH	7.5 ± 0.06^c^	7.2 ± 0.05^b^	5.6 ± 0.03^a^	5.6 ± 0.02^a^	5.7 ± 0.02^a^	5.7 ± 0.01^a^	5.5 ± 0.03^a^
Caecum weight (g)	0.60 ± 0.03^ab^	0.54 ± 0.02^a^	0.66 ± 0.03^b^	0.62 ± 0.04^ab^	0.66 ± 0.03^ab^	0.71 ± 0.04^b^	0.95 ± 0.04^c^
Caecal contents (g)	1.3 ± 0.05^a^	1.58 ± 0.08^ab^	1.50 ± 0.11^ab^	1.24 ± 0.05^a^	1.97 ± 0.06^b^	1.76 ± 0.09^b^	2.43 ± 0.19^c^

^1 ^Means ± SEM, n = 12. Means with superscripts without a common letter differ, P < 0.05.

### Faecal output

Faecal output was increased in the RS- and cellulose-fed rats compared to the rats fed the Control diet (P < 0.001). Forms of RS2 did not increase stool bulk as much as the source with RS3 (i.e. NOVELOSE 330); NOVELOSE 330 was associated with significantly increased faecal output compared to all RS2 treatment groups (P < 0.001) and the Cellulose group (Table 3).

### Caecal parameters

The wet weight of caecal contents was lowest in the Control fed rats and highest in the NOVELOSE 330 (P < 0.001) fed rats, while all other diets showed intermediate weights. The caecal wall weight was significantly higher in the rats fed the NOVELOSE 330 diet (P < 0.001) compared to any other diet (Table 3).

### Effect of the diets on fermentation patterns

The pH was significantly lower in both the caecum and faeces in the rats fed the different RS diets (P < 0.001) compared to rats fed a Control or Cellulose diet (Table 3).

Digesta SCFA concentrations are shown in Table 4. As a generalisation, the SCFA concentrations in the caecum digesta were approximately twice those of the faeces. For both faecal and caecal material, the lowest total and individual SFCA levels were seen in those fed Control and Cellulose diets.

**Table 4:** Effect of experimental diets on SCFA concentrations in caecum and faeces^1^

	**Control**	**Cellulose**	**HYLON VII**	**Hi-maize 1043**	**Hi-maize 240**	**Hi-maize 260**	**NOVELOSE 330**
**Caecal**							
Total SCFA	44.7 ± 3.5^a^	40.0 ± 3.1^a^	62.8 ± 6.9^ab^	78.9 ± 9.4^b^	54.8 ± 5.8^ab^	71.0 ± 8.7^b^	56.5 ± 7.3^ab^
Acetate	28.9 ± 2.5	26.2 ± 2.3	36.6 ± 4.7	41.4 ± 5.3	33.5 ± 4.3	31.4 ± 4.7	40.2 ± 5.7
Propionate	10.7 ± 0.8^a^	8.1 ± 0.8^a^	9.5 ± 1.7^a^	22.3 ± 2.9^c^	12.9 ± 1.2^ab^	16.8 ± 2.2^bc^	10.2 ± 1.1^a^
Butyrate	5.1 ± 0.4^a^	4.4 ± 0.80^a^	15.1 ± 3.1^bc^	12.3 ± 1.7^b^	6.5 ± 1.1^a^	20.3 ± 3.0^c^	4.4 ± 0.8^a^
**Faecal**							
Total SCFA	25.1 ± 3.1^ab^	19.5 ± 2.0^a^	40.5 ± 4.1^bc^	48.2 ± 5.4^c^	44.2 ± 7.9^c^	37.9 ± 2.9^bc^	35.2 ± 4.0^abc^
Acetate	17.3 ± 2.0^ab^	14.4 ± 1.4^a^	24.0 ± 2.7^bc^	28.6 ± 3.6^c^	25.7 ± 2.2^bc^	19.5 ± 2.2^abc^	25.3 ± 3.2^bc^
Propionate	5.0 ± 0.9^a^	3.7 ± 0.4^a^	4.0 ± 0.8^a^	11.1 ± 1.4^b^	4.9 ± 0.6^a^	5.9 ± 0.5^a^	5.9 ± 1.0^a^
Butyrate	2.7 ± 0.4^a^	1.8 ± 0.2^a^	11.5 ± 4.0^b^	6.5 ± 0.9^ab^	3.8 ± 1.3^a^	11.0 ± 2.6^b^	2.5 ± 0.5^a^

^1 ^Means ± SEM, n = 12. Means with superscripts without a common letter differ, P < 0.05.

Caecal total SCFA concentration was highest in rats fed the Hi-maize 1043 and Hi-maize 260 and lowest in those fed the Control fed and cellulose fed rats. Caecal butyrate concentration varied widely between diets and highest levels were observed with the RS2 starches especially HYLON VII and Hi-maize 260; these rats showed a four-fold increase compared to the Control rats. No significant differences were seen between diets for caecal acetate concentration. Caecal propionate concentration was the highest in the Hi-maize 1043 fed rats. Clearly, ratios between SCFA varied between RS starch.

In the faeces, highest total SCFA levels were seen in rats fed Hi-maize 240 and Hi-maize 1043. Butyrate concentration in the faeces, as in caecal digesta, was significantly elevated by the HYLON VII and Hi-maize 260 diets compared to the Control diet. Acetate concentration in the faeces was significantly higher in the Hi-Maize 1043 fed rats compared to the Control fed rats. The Hi-maize 1043 diet was the only diet to significantly elevate propionate concentration.

By considering concentrations in caecal digesta and faeces, it is possible to determine which diets maintained higher concentrations of butyrate along the length of the colon. HYLON VII and Hi-maize 260 stood out significantly from the other diets in this respect.

### Effects of diet on apoptosis and epithelial kinetics in the distal colon

**Figure 1:**
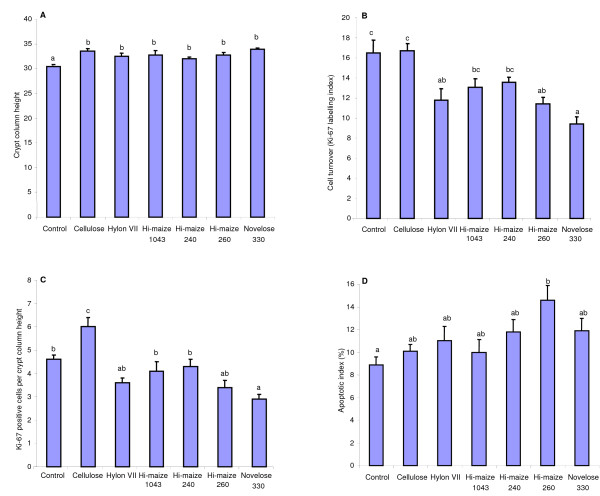
**Effect of experimental diets on colonic epithelial events**. Effect of experimental diets on colonic crypt column height (A), colonic epithelial cell turnover (B), colonic positive Ki-67 cells (C) and AOM-induced apoptosis (D). Means ± SEM, n = 12. Means with superscripts without a common letter differ, P < 0.05.

Crypt column height was significantly different between the different dietary groups (P < 0.001). Atrophy of the colonic crypts was observed in the rats fed the Control diet, compared to those fed the Cellulose and RS diets (Fig. 1A).

Epithelial turnover is assessed from the Ki-67 labelling index and was significantly affected by the different experimental diets (Fig. 1B). The Control and Cellulose groups had significantly higher cell turnover than did Hi-Maize 260, HYLON VII and NOVELOSE 330 groups. Interestingly, cell mass as reflected in crypt column height did not parallel cell turnover. This implies a difference in epithelial proliferation rates. Therefore, we assessed crypt cell proliferation rates from counts of Ki-67-labelled cells per crypt column, as shown in Fig 1C. The Cellulose group had significantly higher Ki-67 counts than all other dietary groups (P < 0.01). Whereas the NOVELOSE 330 group had significantly lower Ki-67 counts than Control, Cellulose, Hi-maize 1043 and Hi-maize 240 groups (P < 0.05).

The apoptotic response to genotoxic damage (AI) in the distal colon was significantly affected by diet (Fig. 1D). The apoptotic response was significantly increased in the Hi-maize 260 fed rats (P < 0.01) when compared to the Control fed rats.

### Relationships between luminal events and epithelial responses

The apoptotic response to genotoxic damage was found to be significantly associated with a number of parameters in caecal digesta and faeces after controlling for the effect of the different diets (Table 5). A significant positive relationship was seen between caecal (r = 0.28, P = 0.004) and faecal butyrate (r = 0.27, P = 0.008) concentrations and the apoptotic response to AOM.

**Table 5:** Correlations between apoptotic index, cell turnover, Ki-67 counts and crypt height in distal colon and selected caecal and faecal parameters^1^

	**Apoptotic index**		**Cell turnover**		**Ki-67 counts**		**Crypt column height**	
	*r*	*P-value*	*r*	*P-value*	*r*	*P-value*	*r*	*P-value*
*Caecum*								
Total SCFA (μmol/g)	0.130	0.19	-0.376	0.005	-0.306	0.02	-.114	0.26
Acetate	0.014	0.89	-0.314	0.02	-0.23	0.09	-0.09	0.37
Propionate	0.071	0.48	-0.196	0.15	-0.16	0.26	-0.16	0.10
Butyrate	0.280	0.004	-0.325	0.02	-0.3	0.03	-0.07	0.46
pH	-0.191	0.052	0.635	0.001	0.57	0.001	-0.08	0.44
*Faeces*								
Total SCFA	0.06	0.56	-0.104	0.45	-0.19	0.18	-0.13	0.21
Acetate	-0.039	0.69	-0.253	0.06	-0.27	0.05	-0.1	0.34
Propionate	0.018	0.86	-0.065	0.64	-0.06	0.64	-0.17	0.10
Butyrate	0.265	0.008	-0.257	0.06	-0.25	0.06	-0.12	0.25
pH	-0.164	0.099	0.610	0.001	0.54	0.001	-0.08	0.41

^1 ^Relationship between caecal, colonic and faecal variables with apoptosis and epithelial kinetics were analysed by Spearman's correlation test.

Cell turnover (Ki-67 labelling index) was negatively correlated with caecal total SCFA (r = -0.38, P = 0.005), caecal acetate (r = -031, P = 0.019) and caecal butyrate (r = -0.325, P < 0.001) but positively correlated with caecal pH (r = 0.63, P < 0.001) and faecal pH (r = 0.61, P < 0.001).

Ki-67-lableld cell counts were negatively correlated with caecal total SCFA (r = -0.31, P = 0.02), caecal butyrate (r = -0.30, P = 0.03), faecal acetate (r = -0.27, P = 0.05), but positively correlated with caecal pH (r = 0.57, P < 0.001) and faecal pH (r = 0.54, P < 0.001).

No significant correlations were observed between crypt column height and any of the luminal variables.

## Discussion

Our findings show that fermentation-dependent luminal events do differ between forms of HAMS, even between starches of the same physico-chemical type, namely RS2. The findings also show that epithelial events are dependent on RS-induced changes in the luminal environment. These significant consequences for epithelial biology might be relevant to risk for colorectal disease including neoplasia.

Increased fermentation, reflected in lowered pH and increased concentrations of SCFA in the digesta, has previously been reported with RS in rats [20,21], pigs [15] and humans [3]. We found high concentrations of SCFA, particularly butyrate with selected RS forms (i.e. Hi-maize 260 and HYLON VII); furthermore both of these maintained the highest butyrate levels along the length of the colon. We have previously shown that maintenance of active fermentation along the length of the colon, including high concentrations of butyrate, is significant in protecting against colorectal cancer development in animal models where, as in humans, cancers predominate in the distal colon [10,11,22].

Plausible explanations that could account for maintenance of high butyrate levels throughout the colon include the RS type, RS amount and/or the dietary fibre content of individual sources of RS and the degree of treatment (specifically hydrothermal treatment). Unfortunately, the present study was unable explain what the critical characteristic of HAMS was that maintained high butyrate levels. It did not appear to be dietary fibre as Hi-maize 260 and HYLON VII which were the best diets for butyrate production are both RS2-type starches, contain similar amounts of RS (46% and 48% respectively) [23] yet the total dietary fibre levels are quite different (60% and 18% respectively) [23]. The hydrothermal treatment also does not seem to have significantly improved fermentation, because HYLON VII is the base starch for the hydrothermal preparation of Hi-maize 260, and it was both of these RS forms that performed the best in terms of SCFA production including butyrate. Quite significant differences in effects on butyrate production were observed between the different RS2-type starches, however, on balance the RS2-type starches tended to perform much better than the single RS3-type starch (ie. Novelose 330). Other factors not measured in the current study that might account for the higher butyrate production could be related to alterations in the colonic microbiota. Certain dietary carbohydrates have been shown to have the capacity to stimulate the growth of particular butyrate producing bacteria directly, or indirectly through stimulation of non-butyrate producing bacteria via metabolic cross-feeding [24]

Butyrate, the physiologically most important SCFA, is produced by anaerobic fermentation of carbohydrate and other substrates in the colonic lumen and is considered to be protective against CRC [25]. In addition to our rodent studies linking butyrate to protection in rodent models [10,11,22] others have also shown that high butyrate producing substrates [26] and delivery of butyrate directly to the distal colonic mucosa have been linked to protection against the initial stages of colon carcinogenesis [27]. Butyrate production has been also been linked to reversing the genetic damage and loss of mucosal barrier in rats induced by feeding higher protein diets [28,29]. In addition to these anti-carcinogenic effects butyrate may also exert anti-inflammatory effects in vivo [30,31].

Other luminal consequences of fermentation were also significantly altered by feeding these HAMS. A lower pH throughout the large bowel was observed. An acidified colon is associated with a decreases risk for CRC cancer [32]. Lower pH values are believed to prevent the overgrowth of pH-sensitive pathogenic bacteria and lower the production of potentially harmful toxic or carcinogenic products in the colon, including secondary bile acids and protein fermentation products (ammonia and phenols) [2,33]. Faecal output was increased by feeding cellulose and all the RS forms. The RS3 'NOVELOSE 330' was the most effective diet at increasing large bowel digesta weight and faecal output; this may be a characteristic of being a retrograded starch.

Luminal events are clearly linked to changes in epithelial biology. Apoptosis is an important innate cellular event for protection against the development of colorectal cancer. This includes removal of cells with genomic instability that have developed during oncogenesis [34] and deletion of cells suffering DNA insult from genotoxic agents such as carcinogens [35]. Enhancement of apoptosis during initiation events increases elimination of mutated cells that might otherwise progress to malignancy [36] and defective apoptotic response is associated with increased risk [37]. In the present study we have examined the relationship between butyrate and the apoptotic response to carcinogen by Spearman correlation procedures using data from individual animals performed regardless of dietary group. We found that butyrate concentration in both the caecum and faeces was positively correlated with AOM-induced apoptosis in the distal colon. This is consistent with earlier reports and confirms an important facilitatory role of butyrate for apoptosis in epithelial homeostasis [18,20]. Production of butyrate by fermentation of RS seems a likely explanation by which RS enhances the apoptotic response to carcinogen-induced genomic lesions. The present study showed that the apoptotic response to the carcinogen was significantly higher in the Hi-maize 260 group compared to the Control group, however Hi-maize 260 was not significantly different from the other RS containing groups. It appears that neither the degree of processing or dietary fibre content was directly related to the apoptotic response rather it was butyrate levels that predicted the apoptotic response

Colonic cell mass is dependent on the presence of RS or dietary fibre as crypt atrophy was observed in the rats fed a diet deprived of RS and dietary fibre. This effect is consistent with our previous studies [38,39] and that of others [40,41]. All of the RS diets (ie. both RS2 and RS3) and the Cellulose diet reversed the atrophy which was observed with the fibre/RS free Control diet. Fermentative production of SCFA is considered to have a trophic affect on the colonic epithelium [2], in the present study cellulose reversed the colonic atrophy independent of SCFA production. Reversal of atrophy by a non-fermentable fibre such as this cellulose is not exactly the same mechanism as with RS, however, because cell turnover and proliferation were affected somewhat differently between cellulose and the RS forms used. Our results also showed reduced colonic cell proliferation (as measured by Ki-67 labelling index) in the rats fed any of the RS containing diets. Increased cell turnover may enhance the risk of mutations which can lead to an increased risk of developing colorectal cancer [42]. Similar reductions in cell proliferation have also been observed in rats fed fermentative substrates like RS [12,16,28] and the carbohydrate oligofructose [43]. It is likely that the increased SCFA resulting from fermentation of starch in the colon contributed to the observed effects on colonic epithelial proliferation.

In conclusion, the present results show that consumption of high amylose maize starches which are rich sources of RS elicit a range of effects in the luminal environment and epithelial biology of the large bowel of rats. These effects differ significantly between different forms of RS and are not necessarily consistent either within a specific physico-chemical type (namely RS2) nor are they obviously predictable from the known characteristic of a RS. On the other hand, generation of high concentrations of distal colonic butyrate is associated with reduced cancer risk, particularly in experimental models and with facilitation of the epithelial apoptotic response to genomic damage. As a consequence, faecal butyrate concentrations might be a useful biomarker of risk for CRC and might provide more information than attempts to ascertain types and amount of RS in the diet (and fibre for that matter). This has implications for human epidemiological and interventional studies trying to further explore the usefulness of RS for protection against CRC. Predicting benefit from apparent dietary composition might not be useful. Furthermore, designing specific interventions with an RS might require a careful consideration of its impact on fermentation events including butyrate in the distal colon.

## Competing interests

RL and GY declare they are recipients of a research grant from National Starch and Food Innovation, but otherwise all authors declare no other potential conflicts of interest.

## Authors' contributions

RL, IB and GY conceived and designed the study. RL and YU carried out animal treatments, biochemical analysis, and data analysis. RL, YU and GY participated in interpretation of results and preparation of the article. All authors read and approved the final version of this manuscript.
